# Microbiological Profiles of the Anatomical Sites of Perforation Peritonitis: A Cross-Sectional Study

**DOI:** 10.7759/cureus.64415

**Published:** 2024-07-12

**Authors:** Samir Deolekar, Robin Patil, Manasi Sawant, Srinivasan P

**Affiliations:** 1 General Surgery, King Edward Memorial Hospital and Seth Gordhandas Sunderdas Medical College, Mumbai, IND; 2 Urology, King Edward Memorial Hospital and Seth Gordhandas Sunderdas Medical College, Mumbai, IND; 3 General Surgery, Grant Government Medical College and Sir J. J. Group of Hospitals, Mumbai, IND

**Keywords:** perforation peritonitis, drug senisitivity, clinical microbiology, ileum, antibiotics sensitivity, peritonitis, perforation

## Abstract

Introduction

One of the most frequent emergencies that a general surgeon deals with is perforation peritonitis. The anatomical site of the perforation, which in turn affects the source of infection, has a major impact on the mortality rate due to perforation peritonitis. Early and suitable antibiotic therapy can be started in the postoperative period with the aid of knowledge about the microbiological profile and sensitivity of peritoneal fluid culture with respect to the anatomical sites of perforation peritonitis.

Methods

A cross-sectional study was conducted from June 2021 to November 2021 where peritoneal fluid samples were collected intraoperatively from patients with perforation peritonitis. This was subjected to culture and sensitivity, and results were analyzed with respect to anatomical sites of perforation.

Results

Forty cases were investigated. The ileum (30%) was the most common site of perforation, followed by the stomach (22.5%), appendix (20%), duodenum (12.5%), caecum (5%), jejunum (5%), transverse colon (2.5%), and rectum (2.5%). *Escherichia coli* (*E. coli*) and *Klebsiella *spp. were the most frequently found organisms in all sites of perforation peritonitis. The most sensitive antibiotics covering all isolated organisms were amikacin and meropenem. Sensitivity to amikacin was found in 85.18% of cases of *E. coli* and 84.6% of cases of *Klebsiella*. Sensitivity to meropenem was found in 76.9% of cases of *E. coli* and 80% of cases of *Klebsiella*.

Conclusion

In patients with perforation peritonitis, the peritoneal fluid cultures did not reflect the major differential normal flora according to the region of the gastrointestinal tract. The most prevalent organism isolated among all the sites of perforation peritonitis was *E. coli*. Antimicrobial activity against organisms isolated from perforation peritonitis patients was significantly demonstrated by aminoglycosides, piperacillin and tazobactam, and meropenem and colistin, with considerable resistance to third-generation cephalosporins.

## Introduction

Surgical peritonitis is one of the most common surgical emergencies in tertiary care centers in India, with most of the patients presenting late in the course of the disease. The mortality rates from intraabdominal infections are largely dependent on the anatomical site of perforation, which in turn influences the source of infection [1, 2]. Several studies have reported a mortality rate of 3%-28% in gastroduodenal perforation, 20%-38% in small bowel perforation, and 20%-45% in cases of large bowel perforation. The most accepted protocol of treatment for patients with secondary peritonitis due to hollow viscus perforation is the resuscitation of the patient and removing the source of contamination as soon as possible, along with the appropriate antimicrobial therapy [3].

Knowledge of the microbial distribution according to the anatomical site of perforation is essential because understanding the regional distribution and characteristics of bacteria will ensure an optimal empirical choice of antibiotic in these patients. It can be obtained by culture of peritoneal fluid obtained intraoperatively [3]. Although there are several guidelines for empirical antibiotics to treat intra-abdominal infections published, the majority of studies on causative bacteria have been done before the 2000s [4-6].

## Materials and methods

Study design

This descriptive cross-sectional study was conducted in the Department of General Surgery at King Edward Memorial Hospital and Seth Gordhandas Sunderdas Medical College, Mumbai, India. The institutional ethics committee (IEC) of King Edward Memorial Hospital and Seth Gordhandas Sunderdas Medical College issued approval (approval number: EC/203/2021).

Study population

All patients who underwent exploratory laparotomy because of perforation peritonitis from June 2021 to November 2021 were part of the study population.

Sample size 

The study had a sample size of 40 participants.

Inclusion criteria

Patients aged 18 years and older and those diagnosed with perforation peritonitis and undergoing emergency exploratory laparotomy were included in the study.

Exclusion criteria

Patients under the age of 18 years and pregnant women were excluded from the study.

Patients and methods

After the application of inclusion and exclusion criteria, patients undergoing exploratory laparotomy for perforation peritonitis were included in the study. Intraoperative findings were confirmed. During routine follow-up visits at two weeks and one month, culture sensitivity reports were recorded. All the data were recorded on pre-approved case record forms. Data were entered in a Microsoft Excel sheet (Microsoft Corp., Redmond, WA) and analyzed to produce results.

Statistical analysis

Data from the case record forms were transcribed into a Microsoft Excel sheet and analyzed using IBM SPSS Statistics software for Windows, version 21.0, (IBM Corp., Armonk, NY). Descriptive data were represented as mean±standard deviation, frequencies, and percentages.

## Results

Age-wise distribution

This study consisted of 40 patients. The most common age group of presentation was 18 to 27 years, followed by 28 to 37 years (Table 1).

**Table 1:** Age-wise distribution of the study group (n = 40)

Age group (in years)	Frequency (n)
18-27	16
28-37	9
38-47	7
48-57	3
58-67	5

Gender-wise distribution

Within the study group, the male-to-female ratio was 3.4:1 (Table 2).

**Table 2:** The distribution of the study group according to gender

Gender	Frequency (n)	Percentage (%)
Female	9	22.5%
Male	31	77.5%
Total	40	100%

Sites of perforation

**Figure 1:**
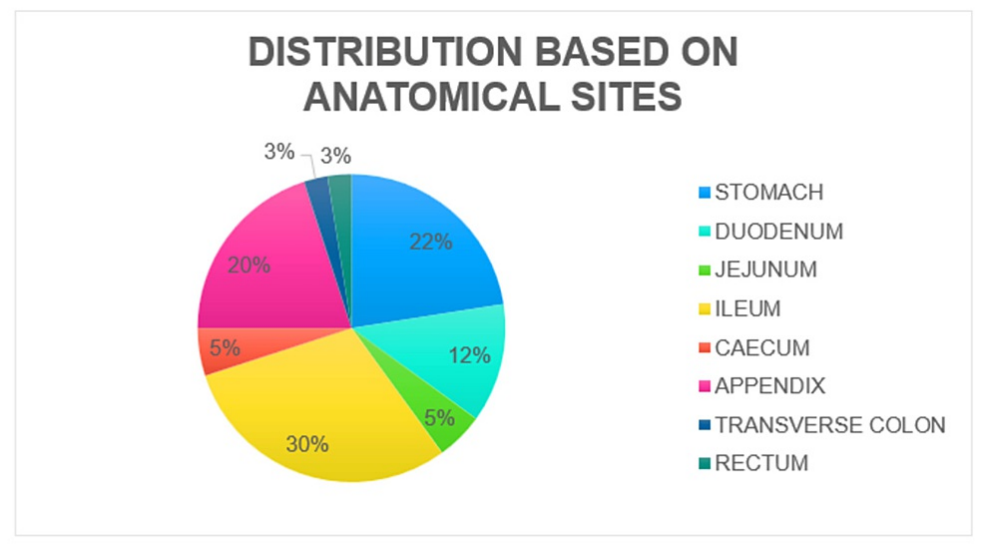
Distribution of study participants based on the anatomical sites of perforation

The ileum (30%) was the most common site of perforation, followed by the stomach (22%), and the appendix (20%) (Figure 1, Table 3).

**Table 3:** Distribution of cases based on the anatomical sites of perforation

Anatomical site of perforation	Frequency (n)	Percentage (%)
Stomach	9	22.5%
Duodenum	5	12.5%
Jejunum	2	5%
Ileum	12	30 %
Caecum	2	5%
Appendix	8	20%
Transverse colon	1	2.5%
Rectum	1	2.5%

Organisms cultured

**Figure 2:**
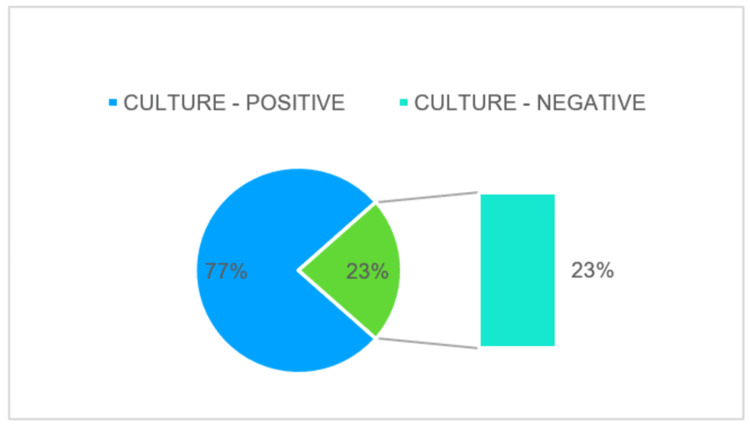
Peritoneal fluid cultures demonstrate both culture positivity and culture negativity.

Among the 40 cases evaluated, culture positivity was found in 31 cases. No growth of organisms was found in nine cases (Figure 2).

Among the positive intraoperative fluid samples, *Escherichia coli *(*E. coli*) was the predominant organism observed (n = 27, 87.09%). Following closely, *Klebsiella pneumoniae* was the second most frequently identified organism (n = 13,41.09%). Additional organisms isolated from the cultures encompassed *Citrobacter *(n = 2, 6.4%), *Acinetobacter *(n = 1, 3.2%), and *Enterococcus *(n = 1, 3.2%).

Stomach

Of the nine cases of stomach perforation, four (44.4%) had positive cultures, and five (55.5%) had negative cultures. Three culture-positive cases had *E. coli* identified; one case had *Acinetobacter *spp., and one case had *Klebsiella *spp. (Table 4).

**Table 4:** Culture reports of cases with stomach perforation

	Number	Percentage (%)
Culture-negative	5	55.5%
Culture-positive	4	44.4%
Organisms		
Escherichia coli	3	75%
*Acinetobacter *species	1	25%
*Klebsiella *species	1	25%

Duodenum

Out of the five cases of duodenal perforation, four cases (80%) had positive cultures, and one case (20%) had negative cultures. Three of the culture-positive cases had *E. coli*, two had *Klebsiella *spp., and one had *Enterococcus *(Table 5).

**Table 5:** Culture reports of cases with duodenal perforation

	Number	Percentage (%)
Culture-negative	1	20%
Culture-positive	4	80%
Organisms		
Escherichia coli	3	75 %
*Klebsiella *species	2	50%
Enterococcus	1	25%

Jejunum

Two cases (100%) of jejunal perforation were found to be culture-positive, out of which two cases had *E. coli* isolated and one case had *Klebsiella *spp. (Table 6).

**Table 6:** Culture reports of cases with jejunal perforation

	Number	Percentage (%)
Culture-negative	0	0
Culture-positive	2	100%
Organisms		
Escherichia coli	2	100%
*Klebsiella *species	1	50%

Ileum

Out of the 12 ileal perforation cases, 10 (83.3%) had positive cultures, and two (16.6%) had negative cultures. Nine cases (of the culture positives) had *E. coli* isolated, five had *Klebsiella* spp., and two had *Citrobacter *(Table 7).

**Table 7:** Culture reports of cases with ileal perforation

	Number	Percentage (%)
Culture-negative	2	16.66 %
Culture-positive	10	83.33%
Organisms		
Escherichia coli	9	90%
*Klebsiella *species	5	50%
Citrobacter	2	20%

Caecum

Two cases (100%) of caecal perforation were culture-positive. Out of which, *E. coli* was isolated in both cases (Table 8).

**Table 8:** Culture reports of cases with caecal perforation

	Number	Percentage (%)
Culture-negative	0	0
Culture-positive	2	100%
Organisms		
Escherichia coli	2	100%

Appendix

Out of the eight cases of appendicular perforation, seven (87.5%) had positive cultures, and one (12.5%) had negative cultures. Six out of the culture-positive cases had *E. coli* isolated, and four had *Klebsiella *spp. (Table 9).

**Table 9:** Culture reports of cases with appendicular perforation

	Number	Percentage (%)
Culture-negative	1	12.5 %
Culture-positive	7	87.5%
Organisms		
Escherichia coli	6	85.7%
*Klebsiella *species	4	57%

Rectum

The single case of rectal perforation was found to be culture-positive; *E. coli* was isolated in the cultures (Table 10).

**Table 10:** Culture reports of cases with rectal perforation

	Number	Percentage (%)
Culture-negative	0	0
Culture-positive	1	100%
Organisms		
Escherichia coli	1	100%

Transverse colon

The single case of colonic perforation was found to be culture-positive; *E. coli* was isolated in the cultures (Table 11).

**Table 11:** Culture reports of cases with colonic perforation

	Number	Percentage (%)
Culture-negative	0	0
Culture-positive	1	100%
Organisms		
Escherichia coli	1	100%

Sensitivity patterns of common antibiotics

Sensitivity Pattern of E. coli

**Figure 3:**
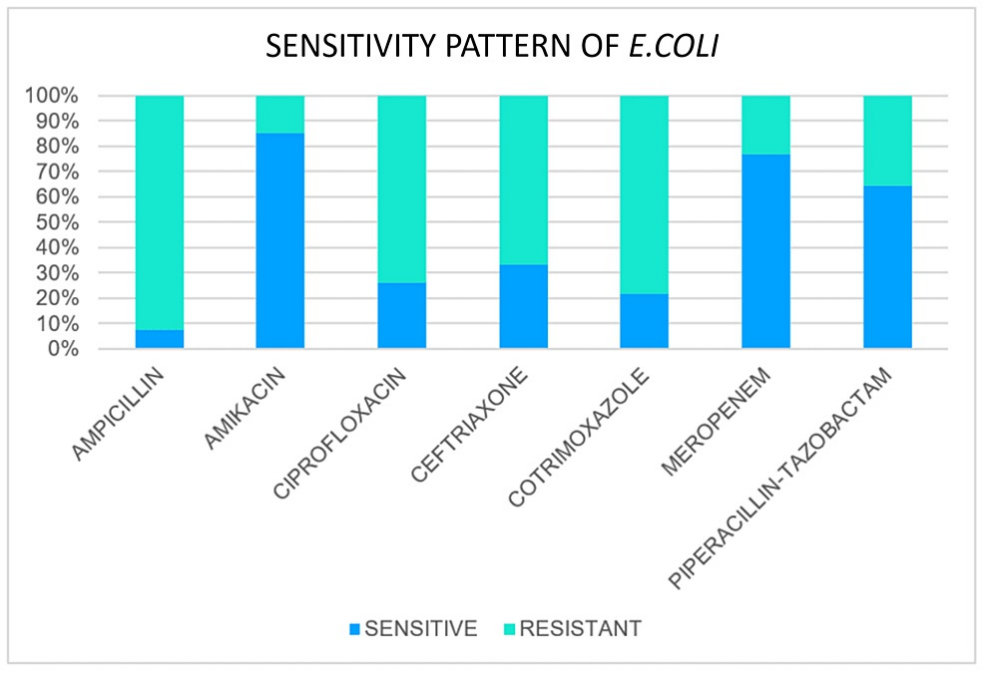
Sensitivity pattern of E. coli *E. coli*: *Escherichia coli *

*Escherichia coli* was isolated in 27 cases. Out of 27 cases, it was found to be sensitive to ampicillin in two cases and resistant in 25 cases. Sensitivity to amikacin was found in 23 cases, and ciprofloxacin was found in seven cases out of the total 27 cases. Out of 24 cases tested for ceftriaxone, sensitivity was found in eight cases. Out of 23 cases tested for cotrimoxazole, sensitivity was found in five cases. Out of 13 cases tested for meropenem, sensitivity was found in 10 cases. Out of 14 cases tested, piperacillin-tazobactam sensitivity was found in nine cases (Figure 3).

Sensitivity Pattern of Klebsiella

**Figure 4:**
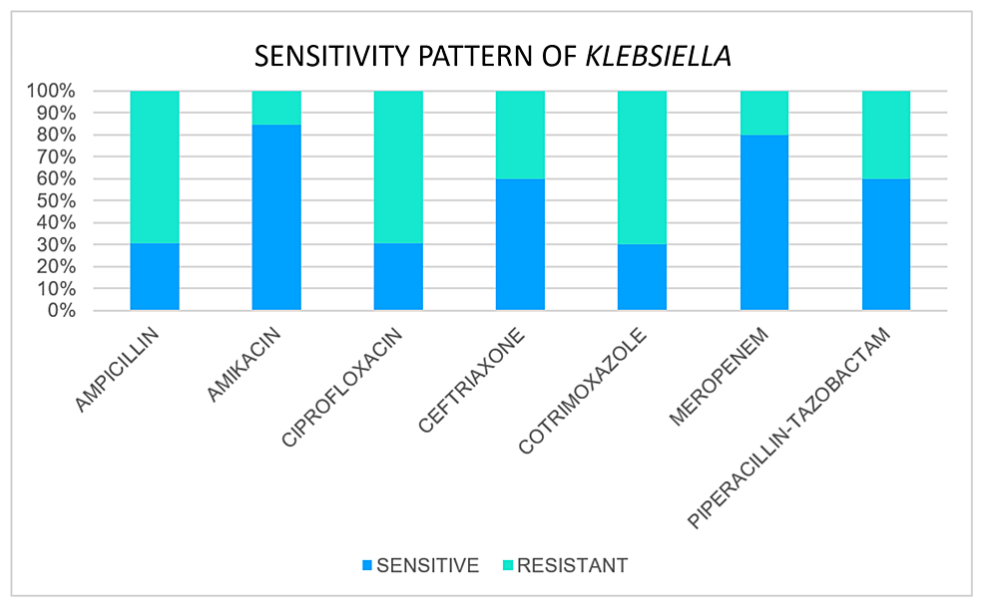
Sensitivity pattern of Klebsiella species

*Klebsiella *was isolated in 13 cases. Out of 13 cases, it was found to be sensitive to ampicillin in four cases and resistant in nine cases. Sensitivity to amikacin was found in 11 cases, and ciprofloxacin was found to be sensitive in four cases out of a total of 13 cases. Out of 10 cases tested for ceftriaxone, sensitivity was found in six cases. Out of 10 cases tested for cotrimoxazole, sensitivity was found in three cases. Out of five cases tested for meropenem, sensitivity was found in four cases. Out of 10 cases tested for piperacillin-tazobactam sensitivity, six cases were found (Figure 4).

Sensitivity Pattern of Citrobacter

**Figure 5:**
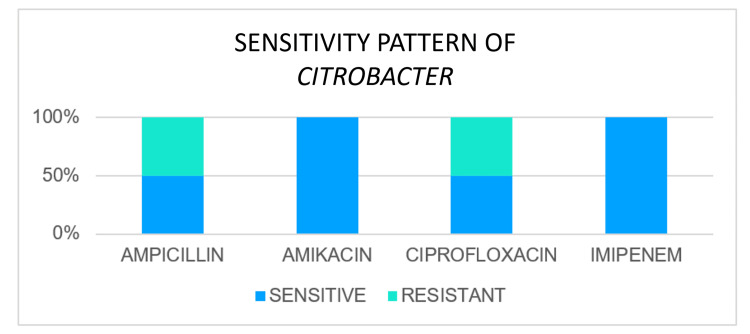
Sensitivity pattern of Citrobacter species

*Citrobacter *was isolated in two cases. Out of two cases, it was found to be sensitive to ampicillin in one case and resistant in another case. Sensitivity to amikacin was found in both cases, and ciprofloxacin was found in one case. Out of one case tested for imipenem, it was found to be sensitive to it (Figure 5).

Sensitivity Pattern of Acinetobacter

*Acinetobacter *was isolated in one case. It was found to be resistant to amikacin, imipenem, and piperacillin. It was found to be sensitive to colistin and tigecycline.

Sensitivity Pattern of Enterococcus

*Enterococcus *was the only gram-positive organism isolated. The isolated organism showed resistance to penicillin and sensitivity to gentamycin, vancomycin, teicoplanin, and linezolid.

## Discussion

This study aimed to assess the microbiological composition of peritoneal fluid cultures in patients diagnosed with perforation peritonitis, analyzing variations based on the anatomical site of perforation. Additionally, it sought to determine the antibiotic sensitivity patterns of the microbes identified in the peritoneal fluid cultures.

Age

The most common age group found to be affected was 18 to 27 years old. The middle age group was affected, which may be due to food habits, lifestyle, and working stress.

Gender

The male-to-female ratio was found to be 3.4:1. In India, perforation peritonitis is more common in males due to the increased incidence of smoking and alcoholism. Male preponderance was found in this study, which is similar to studies conducted by Lohith et al. [1] and Yadav et al. [7]. The most frequent cause of perforation peritonitis in India is gastrointestinal perforation resulting from abdominal tuberculosis, which also exhibits a male preponderance.

Sites of perforation

The ileum was the most frequently perforated site in our study, which is similar to the findings by Lohith et al. [1]. These observations may be attributed to an increased incidence of ileocaecal tuberculosis. According to studies by More et al. [8] and Ravishankar et al. [9], the gastroduodenum was the most frequently perforated site (51% and 94%, respectively). This may be attributed to the high incidence of peptic ulcer disease.

Culture sensitivity 

Similar to the study conducted by Lohith et al. [1], the most frequently isolated organism in gastric perforation cases (44.4%) was *E. coli*. The high stomach acidity, which most organisms find difficult to survive, maybe the cause of the high percentage of culture negativity in gastric perforations.

Culture positivity for duodenal perforation was 80%, with *E. coli* being the most frequently isolated organism, followed by *Klebsiella*. Gram-positive *Enterococcus *was also isolated from one sample. The most frequently isolated organism in cases of jejunal perforation was *E. coli*, with a 100% culture positivity rate. Culture positivity for ileal perforation was 83.3%, with *E. coli* being the most frequently isolated organism, followed by *Klebsiella *and *Citrobacter*. However, the culture positivity rate was significantly higher for jejunal perforations. Gram-negative bacilli (*Enterobacteriaceae*) make up the majority of the flora in the jejunum and ileum normally [1], and these same bacteria were isolated in cultures of perforation peritonitis in both the jejunum and the ileum, with *E. coli* being the predominant isolate from both sites [10].

The most frequently isolated organism in cases of caecal perforation was *E. coli*, with a 100% culture positivity rate. With appendicular perforation, the most frequently isolated organism was *E. coli*, with a culture positivity rate of 87.5%. The most frequently isolated organism in colonic and rectal perforations was *E. coli*, with a 100% culture positivity rate. The higher load of microbial flora in the large intestine is responsible for this high rate of culture positivity. *Escherichia coli* is the most common organism in the aerobic flora. This is consistent with our study's findings, which showed that *E. coli* was primarily isolated from peritoneal fluid cultures in colonic perforations [11, 12].

The bacterial count in the duodenum is 103-106 per gram; in the jejunum and proximal ileum, it is 105-108 per gram; in the lower ileum and caecum, it is 108-1010 per gram; and in the colon, it is 1011 per gram. It has been noted that the bacterial flora in the stomach is almost nil because of the low pH. This demonstrates that the load of microorganisms increases in the gastrointestinal tract from the proximal to the distal regions, and it is consistent with our study that found an increase in culture positivity as the level of perforation moved distally from the stomach to the rectum [1,11].

In our study, the overall most sensitive antibiotics were amikacin and meropenem. Sensitivity to amikacin was found in 85.18% of cases of *E. coli* and 84.6% of cases of *Klebsiella*. Sensitivity to meropenem was found in 76.9% of cases of E. coli and 80% of cases of *Klebsiella*. Other sensitive antibiotics were piperacillin + tazobactam (64.2%. cases of *E. coli* and 60% cases of *Klebsiella*) and ceftriaxone (57% cases of *E. coli* and 60% cases of *Klebsiella*).

The majority of *E. coli* isolates in a study conducted by More et al. [8] were sensitive to amikacin (94%) and ceftazidime (91%). Certain species of *Klebsiella *were sensitive to ciprofloxacin, aminoglycosides, and cephalosporins.

In a study conducted by Ravishankar et al. [9], about 87.5% of *E. coli* were sensitive to ceftriaxone, and about 81.25% were sensitive to ciprofloxacin and amikacin. *Klebsiella *species exhibit a sensitivity of 91.07% to ceftriaxone, with amikacin coming in second at roughly 78% and ciprofloxacin at 73.9%. High levels of resistance to ampicillin and cotrimoxazole were seen in both *E. coli* and *Klebsiella *species. In most cases, organisms were susceptible to ceftriaxone, ciprofloxacin, and amikacin, in that order.

In contrast to other studies, we found that third-generation cephalosporin resistance was significantly higher in our study. It is most likely because the remaining trials were conducted before 2000, a period in which third-generation cephalosporins were widely used and caused the development of resistance. But for aminoglycosides like amikacin, a pattern of sensitivity similar to that seen in earlier research was seen. Thus, in situations with perforation peritonitis, as first-line empirical treatments, we can suggest the prudent use of piperacillin, tazobactam, and aminoglycosides (amikacin). The appropriate antibiotic should then be changed based on the culture report obtained later.

In our study, anaerobic organisms were not isolated at any sites, similar to a study conducted by Ramakrishnaiah et al. [3]. The reason for this is that anaerobes like *Bacterioides *spp. (which are the predominant normal flora present in the colon) are fastidious and slow-growing, and their culture requires strict anaerobic conditions [13, 14].

There is consensus that activity against anaerobes should be provided in most cases [15], and agents lacking such activity are typically combined with antibiotics such as metronidazole. Doses should be adjusted according to renal function and hemodynamic conditions using antibiotics with proven efficacy on susceptibility tests. Theoretically, therapy should last a certain amount of time, but this should be customized for each patient.

## Conclusions

The peritoneal fluid culture of patients with perforation peritonitis did not reflect the major differential normal flora according to the region of the gastrointestinal tract, and *E. coli* was the most prevalent organism isolated in all sites of perforation peritonitis. The third-generation cephalosporins, which are frequently used empirically, are showing signs of growing resistance, according to the antibiotic sensitivity profile. Aminoglycosides still have a high sensitivity profile, nevertheless. Significant antibiotic activity was also demonstrated by piperacillin and tazobactam, meropenem, and colistin against pathogens isolated from perforated peritonitis patients. Systemic antibiotic therapy is the second mainstay of treatment for perforation peritonitis, after source management (such as appendicectomy, perforation closure, resection of gangrenous bowel, and abscess drainage). Early (preoperative) systemic antibiotic therapy can significantly reduce the concentration and growth rates of viable bacteria in the peritoneal fluid, and antibiotic therapy is less effective in the later stages of infection. As part of antibiotic therapy, empiric coverage (effective against common gram-negative and anaerobic organisms) should be initiated as soon as possible. As culture results become available, a step-down approach should then be used to move to narrower-spectrum agents.
